# miR-200b is a key regulator of tumor progression and metabolism targeting lactate dehydrogenase A in human malignant glioma

**DOI:** 10.18632/oncotarget.10301

**Published:** 2016-06-27

**Authors:** Su Hu, Qian Jiang, Dongdong Luo, Lei Zhao, Xin Fu, Yuqin Chen, Xue Song, Lihua Li, Hailin Zhao, Yingfang He, Biao Peng

**Affiliations:** ^1^ Department of Neurosurgery, Cancer Center of Guangzhou Medical University, Guangzhou, Guangdong, 510095, China; ^2^ State Key Laboratory of Respiratory Disease, The 1st Affiliated Hospital of Guangzhou Medical University, Guangzhou, Guangdong, 510120, China; ^3^ Department of Physiology, School of Basic Science, Guangzhou Medical University, Guangzhou, Guangdong, 510182, China; ^4^ Guangdong Provincial Hospital of TCM Breast Department, Guangzhou, Guangdong, 510006, China

**Keywords:** glioma, miR-200b, LDHA, proliferation, metabolism

## Abstract

Lactate dehydrogenase A (LDHA) is involved in various cancers. In this study, we investigated the expression and function of LDHA in glioma. We found that LDHA was up-regulated in glioma samples. Furthermore, we found that overexpression of LDHA promoted proliferation, invasion and glycolysis in glioma cells. Luciferase reporter assays confirmed that LDHA was a direct target of miR-200b. miR-200b was found to be down-regulated in glioma samples, which was inversely correlated with LDHA expression. Repression of LDHA by miR-200b suppressed the glycolysis, cell proliferation and invasion of glioma cells. These results provide evidence that miR-200b acts as a tumor suppressor in glioma through the inhibition of LDHA both *in vitro* and *in vivo*. Targeting LDHA through miR-200b could be a potential therapeutic strategy in glioma.

## INTRODUCTION

Malignant glioma is the most common primary malignant brain tumor in adults [1]. Inspite of recent advances in surgery and adjuvant treatments, the prognosis of glioma is still poor [2]. Therefore, understanding the key regulatory mechanisms of glioma is important to develop novel and effective therapeutic strategies for glioma.

MicroRNAs (miRNAs) are endogenous, non-coding, single-stranded 19- to 25-nucleotide RNAs that regulate the expression of target genes post-transcriptionally [3]. Numerous studies have shown that miRNAs play vital roles in tumorigenesis, progression, diagnosis, prognosis, and therapy [4, 5]. Abnormal expression of miRNAs has been reported in many cancers, including glioma and its aggressive glioblastoma subtype [6–8]. Recently, dysregulation of miR-200 family members, miR-200b especially, has been reported in various human cancers [9, 10]. It's been reported that miR-200b acts as an important regulator in cell cycle, epithelial-to-mesenchymal transition (EMT) and chemosensitivity [11–14]. We reported previously that miR-200b suppressed cell growth in human malignant glioma through directly targeting CREB1 [15]. But how miR-200b functions in glioma metabolism is still unclear.

Lactate dehydrogenase A (LDHA) is an enzyme that plays a vital role in metabolism in cancers [16–19]. It has been reported to correlate with clinicopathologic features and survival outcome of multiple cancers [20–23]. Expression of LDHA is regulated by several oncogenes and deacetylases, such as MYC, HIF-1α [24, 25]. These findings indicated that LDHA could be a novel therapeutic target.

Here, we investigated the expression pattern and function of LDHA in glioma and explored the possible correlation between miR-200b and LDHA in glioma. Our findings may provide significant evidence regarding miR-200b as an important tumor suppressor in glioma through targeting LDHA.

## RESULTS

### LDHA is up-regulated and promotes cell proliferation and invasion in glioma

**Figure 1:**
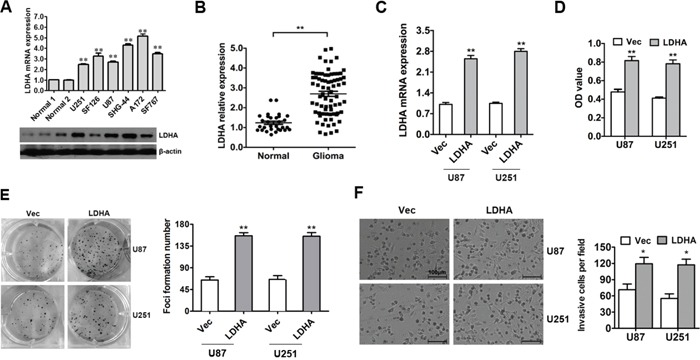
LDHA is up-regulated and promotes cell proliferation and invasion in glioma **A.** The expression of LDHA in glioma cell lines was assessed using qRT-PCR and western blot. β-actin was used as an endogenous normalizer. **B.** The expression level of LDHA in 73 glioma samples (Glioma) and 30 unmatched normal cerebrum samples (Normal) were assessed using qRT-PCR. β-actin was used as an endogenous normalizer. **C.** U87 and U251 cells were transfected with LDHA-expression vector or control vector. The transfection was successful. **D.** Cell viability was determined by MTT assay performed 48 hours after transfection. **E.** Cell growth capacity *in vitro* was assessed by colony formation assay. **F.** Cell invasion capacity was assessed by transwell assay. All of the data are shown as the means ± s.e.m. * *P* < 0.05, ** *P* < 0.01.

To evaluate the expression pattern of LDHA in glioma, qRT-PCR and western blot were performed. The results showed that LDHA was up-regulated in glioma compared to normal samples (Figure 1A). Then we detected the expression level of LDHA in 73 glioma samples and 30 unmatched normal cerebrum samples and found that the expression level of LDHA in glioma was significantly higher than that in normal samples (Figure 1B). Futher we checked the correlation between expression of LDHA and its clinicopathological parameters in that 73 glioma samples. We found that high expression of LDHA was correlated with higer glioma histopatholgy grade(*P*=0.004, Table 1).

**Table 1:** Analysis of the correlation between expression of LDHA and its clinicopathological parameters in 73 glioma samples

Viable	Cases	LDHA		
		low	high	*P* value
Age (years)				
≤45	40	15	25	0.451
>45	33	11	22	
Gender				
Male	37	13	24	0.562
Female	36	13	23	
Glioma histopatholgy				
Grade I-II	26	15	11	0.004*
Grade III-IV	47	11	36	

* statistically significant (*P* < 0.05).

To determine the functions of LDHA in glioma tumorigenesis, LDHA-expressing vector and control vector were used. The alteration of LDHA was confirmed by qRT-PCR (Figure 1C). The effects of LDHA alteration on cell viability and growth were evaluated in glioma cells using MTT and colony formation assays. The results indicated that overexpression of LDHA in U87 and U251 cells markedly increased the viability of glioma cells and promoted cells toform much more colonies than the control group (Figure 1D & 1E). These results demonstrate that LDHA promotes cell proliferation of glioma cells.

To further assess the impact of LDHA on cellular invasion in glioma cells, U87 and U251 cells were transfected with LDHA-expressing vector or control vector and then the transwell assay was performed. The results showed that the ectopic expression of LDHA significantly enhanced cell invasion capacity of U87 and U251 cells compared with the control group (Figure 1F).

Taken together, these results indicated that LDHA was up-regulated and promoted cell proliferation and invasion in glioma.

### LDHA promotes glycolysis in glioma cells

**Figure 2:**
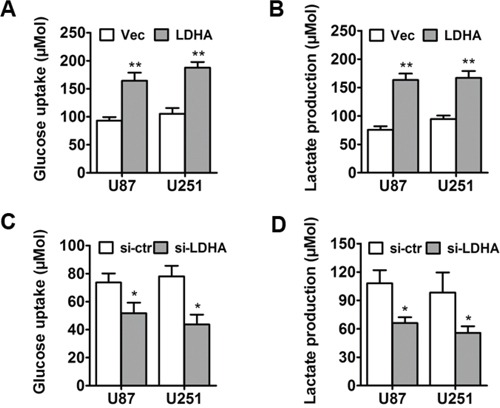
LDHA promotes glycolysis in glioma cells **A.** U87 and U251 cells were transfected with LDHA-expression vector or control vector. After the transfection, the level of glucose uptake was measured. **B.** After the transfection, the level of lactate production was measured. **C.** U87 and U251 cells were transfected with si-LDHA vector or control vector. After the transfection, the level of glucose uptake was measured. **D.** After the transfection, the level of lactate production was measured. All of the data are shown as the means ± s.e.m. * *P* < 0.05, ** *P* < 0.01.

To assess the metabolic effects of LDHA in glioma cells, control vector or LDHA-expressing vector was transfected into U87 and U251 respectively. Then differences in metabolic parameters were examined and we found that overexpression of LDHA largely promoted aerobic glycolysis in glioma cells, e.g., increased glucose uptake and lactate production (Figure 2A and 2B). And when we knocked down LDHA, the cells showed decreased glucose uptake and lactate production (Figure 2C & 2D). These results indicated that LDHA promoted cell glycolysis in glioma.

### LDHA is a direct target of miR-200b and is negatively correlated with miR-200b in glioma

**Figure 3:**
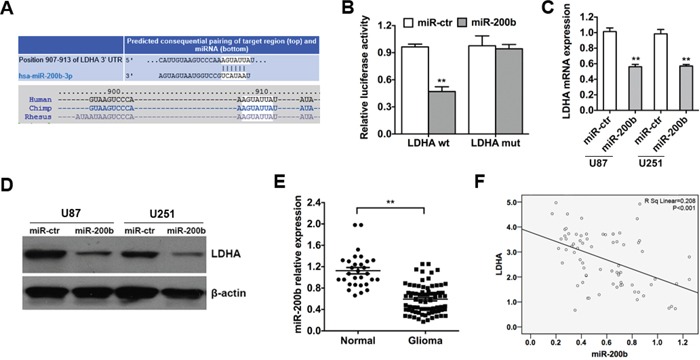
LDHA is a direct target of miR-200b and is negatively correlated with miR-200b in glioma **A.** The predicted binding site of miR-200b on LDHA mRNA is shown. **B.** Luciferase assay on U251 cells co-transfected with miR-200b mimics or scramble control and a luciferase reporter containing the full length of LDHA 3′-UTR (LDHA-wt) or a mutant (LDHA-mut) in which the first five nucleotides of the miR-200b binding site were mutated. An empty luciferase reporter construct was used as a negative control. Luciferase activities were measured 48 hours post-transfection. miR-200b markedly suppressed luciferase activity in LDHA-wt reporter constructs. **C.** U87 and U251 cells were transfected with miR-200b mimics or scramble control, and the level of LDHA mRNA was evaluated by qRT-PCR. β-actin mRNA was used as an endogenous normalizer. **D.** After the tramsfection, the level of LDHA protein was measured by western blot. β-actin was used as an endogenous normalizer. **E.** The expression levels of miR-200b in 73 glioma samples and 30 unmatched normal cerebrum samples were assessed using qRT-PCR. U6 snRNA was used as an endogenous control. **F.** qRT-PCR showed that mRNA levels of LDHA were inversely related to the expression of miR-200b in 73 glioma samples. All of the data are shown as the means ± s.e.m. ** *P* < 0.01.

To identify miRNAs that directly bind to the 3′-UTR of LDHA, we used the mRNA target-predicting algorithms (TargetScan, PicTar and miRanda) and a miR-200b-binding site was found in the 3′-UTR of LDHA (Figure 3A). To verify whether LDHA is a direct target of miR-200b we performed luciferase reporter assays in glioma cell line U251. The relative luciferase activity of U251 cells transfected with the wild-type 3′-UTR of LDHA and miR-200b mimics exhibited an approximately 50% reduction relative to control group (Figure 3B). Additionally, mutation of the putative miR-200b sites in the 3′-UTR of LDHA abrogated the luciferase response to miR-200b (Figure 3B).

To further confirm that LDHA is a direct target of miR-200b, qRT-PCR and western blot analyses were performed to detect whether the expression of LDHA was regulated by miR-200b. The results showed a notable reduction of the mRNA and protein levels of LDHA in cells transfected with miR-200b mimics compared with control group (Figure 3C & 3D).

To test whether the regulations described above for glioma cell lines are also clinically relevant, qRT-PCR was used to detect the expression level of miR-200b in 73 glioma samples and 30 unmatched normal cerebrum samples as described before. Indeed, we found that expression levels of miR-200b in normal samples were higher compared to glioma samples (Figure 3E). Moreover, we analyzed the correlation between the level of LDHA and miR-200b in 73 glioma samples, and detected a negative correlation between them (Figure 3F).

Taken together, these results indicated that LDHA was a direct target of miR-200b and was negatively correlated with miR-200b in glioma.

### LDHA-induced glycolysis, cell proliferation and invasion can be inhibited by miR-200b

**Figure 4:**
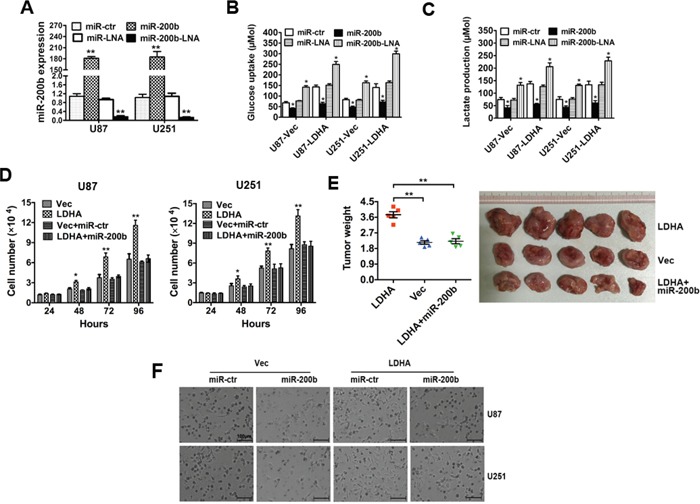
LDHA-induced glycolysis, cell proliferation and invasion can be inhibited by miR-200b **A.** U87 and U251 cells were transfected with miR-200b mimics, miR-200b-LNA or respective control. The alteration of miR-200b was confirmed by qRT-PCR **B.** U87 and U251 cells were transfected with control vector or LDHA-expression vector followed by miR-200b mimics, miR-200b-LNA or respective control. After transfection the level of glucose uptake was measured. **C.** U87 and U251 cells were transfected as described before. The level of lactate production was measured after transfection. **D.** U87 and U251 cells were transfected with control vector, LDHA-expression vector, control vector + scrambled control or LDHA-expression vector + miR-200b mimics respectively. The number of cells was counted. **E.** U87 and U251 cells transfected with LDHA-expression vector, control vector or LDHA-expression vector + miR-200b mimics were injected subcutaneously into nude mice (five in each group). After 28 days, the mice were sacrificed, and necropsies were performed. Then the tumors were weighed. **F.** U87 and U251 cells were transfected with control vector or LDHA-expressing vector followed by scramble control or miR200b mimics. Cell invasion capacity was assessed by transwell assay. All of the data are shown as the means ± s.e.m. * *P* < 0.05, ** *P* < 0.01.

The above results prompted us to validate that miR-200b could indeed inhibit glycolysis and cell proliferation of glioma by targeting LDHA. For this purpose, U87 and U251 cells were transfected with control vector or LDHA-expression vector followed by miR-200b mimics, miR-200b-LNA or respective control. The alteration of miR-200b was confirmed by qRT-PCR (Figure 4A). The results show that increased glycolysis induced by LDHA could be repressed by miR-200b, leading to decreased glucose uptake (Figure 4B) and lactate production (Figure 4C). And when we knocked down miR-200b, cell glycolysis was further improved.

Then we explored whether miR-200b could inhibit cell proliferation through targeting LDHA. U87 and U251 cells were transfected with control vector, LDHA-expressing vector, LDHA-expressing vector + miR-200b mimics or control vector + scramble control respectively. The results show that increased cell proliferation induced by LDHA could be repressed by miR-200b (Figure 4D).

Moreover, we perform xenograft experiments to further investigate the biologic function of miR-200b and LDHA in tumor growth. U251 cells were transfected with LDHA-expressing vector, control vector or LDHA-expressing vector + miR200b mimics. The results showed that ectopic expression of LDHA in U251 cells led to a significant increase in tumor size and tumor weight and transfection of miR200b could abrogate this effect caused by LDHA (Figure 4E).

To further investigate the impact of miR-200b on LDHA in cellular invasion, transwell assay was performed. U87 and U251 cells were transfected with control vector or LDHA-expressing vector followed by scramble control or miR200b mimics. The results showed that the enhanced U87 and U251 cell invasion capacity induced by ectopic expression of LDHA could be abrogated by miR-200b (Figure 4F).

Taken together, these results indicated that LDHA-induced glycolysis, cell proliferation and invasion can be inhibited by miR-200b in glioma cells.

## DISCUSSION

As the most common primary malignant brain tumor in adults, the prognosis of malignant glioma still remains poor. The past few decades have witnessed significant improvements in treatment of glioma, but there still lack more specific and effective strategies.

The roles miRNAs play in cancers have been widely investigated. miRNAs influence cell proliferation, differentiation and metabolism by targeting specific genes [26–28]. miR-200b, like the other members of the miR-200 family, is found to be dysregulated in multiple cancers. Moreover, increasing evidence has demonstrated that the dysregulation of miR-200b largely influences cell proliferation, cell cycle, cell invasion, EMT and chemoresistance of cancer cells [11–14, 29–31]. Thus, miR-200b might be a new weapon to better treat glioma.

Due to the high energy demand and low ATP-generating efficiency, reprogramming of energy metabolism is very common in cancer cells [32–35]. In order to support rapid cell growth, cancer cells have to increase glucose uptake and metabolic intermediates. Aberrant tumor metabolism, which supports tumor cells' energy requirements, has been shown to promote the progression and metastasis of tumor. Among all the glycolytic enzymes, LDH is of great importance to maintain high glycolysis rate. And LDHA is reported to be up-regulated in many cancers [16, 17, 19–21, 23].

Here, we investigated the expression and function of LDHA and the regulation of miR-200b to LDHA in glioma. Firstly, LDHA was found to be up-regulated in glioma cell lines and samples (Figure 1A & 1B). *In vitro* studies showed that LDHA had a positive effect on cell proliferation (Figure 1D & 1E), invasion (Figure 1F) and glycolysis (Figure 2). Then we performed luciferase activity assays, qRT-PCR, western blot assays to confirm that LDHA indeed was targeted by miR-200b (Figure 3B, 3C & 3D). Moreover, we observed a down-regulation of miR-200b in glioma samples, which was inversely correlated with LDHA levels (Figure 3E & 3F). Finally, when we overexpressed miR-200b in glioma cells, the increased cell glycolysis, proliferation and invasion caused by LDHA were inhibited (Figure 4).

Taken together, these results demonstrate that LDHA acts as a tumor promoter in glioma and can be targeted by miR-200b. Inhibiting LDHA through miR-200b could be a promising strategy in treating glioma.

## MATERIALS AND METHODS

### Cell lines and culture

Human glioma cell lines U87, U251, SF126, SHG-44, A712 and SF767 were obtained from the Chinese Academy Medical Science (Beijing) and grown in RPMI-1640 medium supplemented with 10% fetal bovine serum (FBS) and penicillin-streptomycin. All cells were maintained at 37°C in 5% CO_2_ and 95% air.

### Patient samples

We obtained frozen tissue samples of 73 gliomas and 30 normal brain tissues from the Accessory Cancer Center of Guangzhou Medical University (Guangzhou, Guangdong, China) diagnosed between March 2009 and March 2014. Tumor samples were diagnosed by 2 pathologists who were blinded to patient data using the World Health Organization (WHO) system. Clinical data, including gender, age, follow-up, and outcome, were obtained from the medical records. This study was approved by the Ethics Committee of Guangzhou Medical University. All patients provided written informed consent in compliance with the code of ethics of the World Medical Association (Declaration of Helsinki). Eligible patients were recruited from Accessory Cancer Center of Guangzhou Medical University.

### Quantitative RT-PCR analysis (qRT-PCR)

Total RNAs were extracted with TRIzol reagent (Invitrogen). For the detection of LDHA mRNA, cDNA was synthesized from 1 ug of total RNA using the reverse reaction kit according to the manufacturer's instructions (Promega). β-actin was amplified in parallel as an internal control. The primers were: β-actin forward: 5′-AGCGAGCATCCCCCAAAGTT-3′ and reverse: 5′-GGGCACGAAGGCTCATCATT-3′. LDHA forward: 5′-TTGGTCCAGCGTAACGTGAAC-3′ and reverse: 5′-CCAGGATGTGTAGCCTTTGAG-3′. For miR-200b, reverse transcription and qRT-PCR reactions were performed using a qSYBR-green-containing PCR kit (GenePharma, Shanghai, China). U6 snRNA was used as an endogenous control for miRNA detection. The expression of each gene was quantified by measuring cycle threshold (Ct) values and normalized using the 2^−ΔΔCt^ method relative to U6 snRNA or β-actin.

### MTT assay

U87 and U251 cells were seeded in a 96-well plate at 5,000 cells per well one day prior to transfection. The cells were transfected with LDHA-expressing vector or control vector. MTT assay was used to determine cell viability 48 hours after transfection. The absorbance at 570 nm was measured with an uQuant Universal Microplate Spectrophotometer (BioTek, Winooski, VT, U.S.).

### Colony formation assay

U87 and U251 cells were transfected with LDHA-expressing vector or control vector. After transfection, U87 and U251 cells were counted and seeded in 6-well plates (in triplicate) at 150 cells per well. Culture medium was replaced every three days. Colonies were counted only if they contained more than 50 cells, and the number of colonies was determined at the sixth day after seeding.

### Transwell assay

Transwell assay was used to determine the invasion capacity of glioma cells. Briefly, cells were seeded onto the basement membrane matrix present on the insert of a 24-well culture plate (EC matrix, Chemicon, Temecula, CA, USA). Fetal bovine serum was added to the lower chamber as a chemoattractant. After 48 hours, the non-invading cells and the EC matrix were gently removed with a cotton swab. Invasive cells located on the lower side of the chamber were stained with crystal violet, counted and imaged.

### Measurement of glucose consumption and lactate production

LDHA-expressing vector, si-LDHA or respective control vector was transfected into glioma cell lines U87 and U251. Cell culture media were collected 48 hours after the transfection. Glucose uptake and lactate production were measured using Amplex^®^ Red Glucose/Glucose Oxidase Assay Kit (Invitrogen, USA) and lactate assay kit (Sigma, USA) respectively. The results were normalized on the basis of total cellular protein amounts.

### Luciferase assay

The full-length of LDHA 3′-UTR was cloned into the Sacl and Mlul sites of the pMIR-REPORT luciferase vector (Ambion, Austin, TX, U.S.) using PCR generated fragment. A LDHA-mut vector in which the first five nucleotides complementary to the miR-200b seed-region were mutated by site-directed mutagenesis (Stratagene) served as a mutant control. LDHA-wt or LDHA-mut were co-transfected with miR-200b mimics or the scramble control into U251 cells. The pMIR-REPORT β-galactosidase control vector was transfected and served as a control. Luciferase activity was measured in cell lysates 48 hours after transfection using a dual-light luminescent reporter gene assay kit (Applied Biosystems). Results were normalized against β-galactosidase activity.

### Western blotting

Western blotting was carried out as described previously [36]. Anti-LDHA antibody and Anti-β-actin antibody were both obtained from Santa Cruz Biotechnology.

### Cell proliferation assay

U87 and U251 cells were transfected with control vector, LDHA-expressing vector, control vector + scramble control or LDHA-expressing vector + miR-200b mimics. After transfection, cells were plated into six-well plates at the desired cell concentrations. Cell numbers were estimated by trypsinizing the cells. Analysis was performed in triplicate with a Coulter counter (Beckman Coulter, Fullerton, CA, U.S.) at the indicated points in time.

### Mouse xenograft model

A total of 1 × 10^7^ U251 cells infected with LDHA-expressing vector, control vector or LDHA-expressing vector + miR-200b mimics were inoculated subcutaneously into the dorsal flanks of nude mice (five in each group). After 28 days, the mice were sacrificed, necropsies were performed, and the tumors were weighed. All of the animal procedures were performed in accordance with institutional guidelines.

### Statistical analysis

Two-tailed Student's unpaired t-tests were used to evaluate statistical significance. Spearman's correlation tests were used to evaluate the pair-wise expression correlation between miR-200b and LDHA. Data are expressed as means ± s.e.m. *P*<0.05 was considered statistically significant.
